# Fish HERC7: Phylogeny, Characterization, and Potential Implications for Antiviral Immunity in European Sea Bass

**DOI:** 10.3390/ijms25147751

**Published:** 2024-07-15

**Authors:** Yulema Valero, Elena Chaves-Pozo, Alberto Cuesta

**Affiliations:** 1 Immunobiology for Aquaculture Group, Department of Cell Biology and Histology, Faculty of Biology, University of Murcia, 30100 Murcia, Spain; yuvalero@um.es; 2 Centro Oceanográfico de Murcia, Instituto Español de Oceanografía (COMU-IEO), CSIC Carretera de la Azohía s/n, Puerto de Mazarrón, 30860 Murcia, Spain; elena.chaves@ieo.csic.es

**Keywords:** HERC proteins, HERC7, European sea bass, antiviral response, betanodavirus

## Abstract

E3 ubiquitin ligases, key components of the ubiquitin proteasome system, orchestrate protein degradation through ubiquitylation and profoundly impact cellular biology. Small HERC E3 ligases (HERC3-6) have diverse functions in mammals, including roles in spermatogenesis, protein degradation, and immunity. Until now, only mammals’ HERC3, HERC5, and HERC6 are known to participate in immune responses, with major involvement in the antiviral response. Interestingly, an exclusive HERC7 has been characterized in fish showing great molecular conservation and antiviral roles. Thus, this study identifies and characterizes the *herc7* gene in the European sea bass teleost. The European sea bass *herc7* gene and the putative protein show good conservation of the promoter binding sites for interferons and the RCC1 and HECT domains characteristic of HERC proteins, respectively. The phylogenetic analysis shows a unique cluster with the fish-exclusive HERC7 orthologues. During ontogeny, the *herc7* gene is expressed from 3 days post-fertilization onwards, being constitutively and widely distributed in adult tissues. In vitro, stimulated leucocytes up-regulate the *herc7* gene in response to mitogens and viruses, pointing to a role in the immune response. Furthermore, sea bass *herc7* expression is related to the interferon response intensity and viral load in different tissues upon in vivo infection with red-grouper betanodavirus (RGNNV), suggesting the potential involvement of fish HERC7 in ISGylation-based antiviral activity, similarly to mammalian HERC5. This study broadens the understanding of small HERC proteins in fish species and highlights HERC7 as a potential contributor to the immune response in European sea bass, with implications for antiviral defense mechanisms. Future research is needed to unravel the precise actions and functions of HERC7 in teleost fish immunity, providing insights into direct antiviral activity and viral evasion.

## 1. Introduction

E3 ubiquitin ligases are pivotal components of the cellular ubiquitin proteasome system responsible for protein degradation, where they play a significant role in the final protein expression and control every aspect of the cellular biology of eukaryotic cells [1,2,3]. Based on variations in both structure and function, E3 ubiquitin ligases can be broadly categorized into four distinct types: homologous to the E6-associated protein C-terminus (HECT), U-box, RING-finger, and RING-between RING-RING (RBR) [4]. HECT ligases share the homologous catalytic HECT domain, in the C-terminus, while the binding sites for target substrates are located in the N-terminus [4]. This type is categorized into three distinct subfamilies: the Nedd4, the HERC, and different HECT ligases [4,5]. The main feature of the HERC subfamily is the presence of the HECT domain and one or more regulator of chromosome condensation 1 (RCC1)-like domains (RLD), used to divide this subfamily into large (HERC1 and HERC2) and small HERCs (HERC3 to HERC6) [5]. HERC proteins are quite well characterized in mammals and exert broad tissue distribution [6].

Mammalian small HERC proteins show remarkable functions in spermatogenesis, protein degradation, cell signaling, mitosis, tumor inhibition, and immunity (for a review, see [6]). Human (h)HERC4 and hHERC6 have been mostly related to spermatogenesis and male infertility while hHERC3 and hHERC5 (and mouse (m)HERC6) have been unequivocally related to immune responses and inflammation [7,8,9,10], with hHERC5 being highly expressed in testis [6]. In this regard, HERC3 seems to regulate the inflammatory response triggered by NF-κB through its RelA subunit [7]. Strong evidence demonstrates the role of hHERC5 (and mHERC6) in antiviral immunity. Thus, the stimulation of interferon (IFN) resulted in the up-regulation of hHERC5 mRNA levels while the IFN pathway was highly disrupted when hHERC5 or mHERC6 was knocked down [8,9,10,11]. Interestingly, the overexpression of hHERC5 conferred antiviral activity against several viruses [12,13]. In summary, the antiviral role might be mainly carried out by hHERC5, followed by hHERC3 and hHERC4, and inexistent for hHERC6 [8,9,10,13]. hHERC5 serves as the main E3 ubiquitin ligase during viral infections as it is recruited by a complex formed by a target protein and the IFN-stimulated gene (ISG) 15 protein to catalyze their covalent attachment through the ISGylation process [8,10,11,14]. Therefore, HERC5 shows antiviral activity through the ISGylation of viral proteins, reducing viral replication and infectivity [15]. For example, hHERC5 catalyzes the ISGylation of the non-structural NS1 protein of influenza virus, human papilloma virus L1 capsid protein, and human immunodeficiency virus type I Gag enzyme [15,16,17]. In addition, hHERC5 also reduces the ISGylation of IFN regulatory factor 3 (IRF3), promoting the antiviral immune response [9].

Evolutionally, small HERC proteins might have a marine origin and are found in fish [13], though limited information regarding their functions in this vertebrate clade exists. Only a few available transcriptional studies have identified the *herc4* gene during viral infections [18,19,20]. However, deep genomic analyses pointed to the lack of orthologues to mammalian HERC5 and HERC6 in many fish species due to gene loss, while highlighting the presence of a non-mammalian and exclusive fish HERC protein: HERC7 [21,22]. The functional characterization of crucian carp (*Carassius auratus*) and zebrafish (*Danio rerio*) HERC7 has linked it with the antiviral response [21,22]. In both cases, *herc7* mRNA has been up-regulated concomitantly to viral infections but the protein’s overexpression resulted in the inhibition of the IFN response, resulting in higher viral yields. This issue distinguishes the role of fish HERC7 from that of hHERC5. Regarding HERC7’s underlying molecular mechanisms, fish HERC7 overexpression results in the activation of IRF3 but the reduction in most of the IFN pathway mediators (RIG-I, MDA5, MAVS, MITA, IRF, and IFN) [21]. Interestingly, crucian carp HERC7 shows E3 ubiquitin ligase activity, residing in the HECT domain, but this is not mandatory to inhibit the IFN response. In fact, HERC7 favors MITA and MAVS degradation through the proteasome degradation pathway but does not perform their direct ubiquitination, suggesting the implication of other mediators that merit clarification [21]. Interestingly, the overexpression of crucian carp *herc7* gene promotes the downregulation of *irf7* mRNA levels [21], while the overexpression of zebrafish *herc7c* reduces the amount of STING, MAVS, and IRF7 proteins [22], in both cases down-regulating the type I IFN response against viruses. In addition, zebrafish HERC7c shows the potential for ubiquitinylation, similarly to crucian carp HERC7, but not for ISGylation [21,22]. The current understanding of small HERC proteins in fish reveals significant gaps, particularly for HERC7. There is a lack of knowledge on the molecular mechanisms by which HERC7 operates, especially its indirect role in the proteasome degradation pathway and its unique impact on the IFN response. Additionally, the evolutionary aspects, such as the gene loss of HERC5 and HERC6 in fish and the presence of a fish-specific HERC7, are not well understood. Comparative analyses with mammalian HERC proteins are needed to highlight species-specific adaptations and functional differences. Overall, more in-depth functional characterizations, mechanistic studies, and evolutionary investigations would be required to bridge these gaps.

This work aims to characterize a gene encoding for a small HERC protein identified in a transcriptome of European sea bass (*Dicentrarchus labrax*), one of the top 10 most relevant fish species for world marine and coastal aquaculture as well as for Mediterranean aquaculture facilities [23]. The in silico study revealed that the sequence firstly identified as probable *herc4* certainly codifies for the putative HERC7 protein. It clusters in the phylogenetic tree with fish HERC7 orthologues and far from other fish and mammalian small HERCs. Afterwards, the pattern of expression of the European sea bass *herc7* gene was investigated by real-time PCR in naïve tissues; in in vitro-stimulated leucocytes and the *Dicentrarchus labrax* brain-1 (DLB-1) cell line; in in vivo fish infection with red grouper betanodavirus (RGNNV), since European sea bass is highly susceptible to this nodavirus strain [24]; and in vaccinated fish, with the objective of clarifying its potential involvement in the antiviral response of European sea bass. In addition and based on the role of mammalian HERC proteins in reproduction and male infertility and the ability of RGNNV to colonize fish gonad, we also investigated the *herc7* gene in the European sea bass testis infected by RGNNV in vivo and in vitro. By characterizing the European sea bass HERC7 gene, in silico and under in vitro and in vivo conditions, this research bridges critical gaps in knowledge, paving the way for further comparative and functional analyses that could lead to targeted applications in aquaculture and fish health management.

## 2. Results

### 2.1. Identification and In Silico Analysis of European Sea Bass HERC7

**Figure 1:**
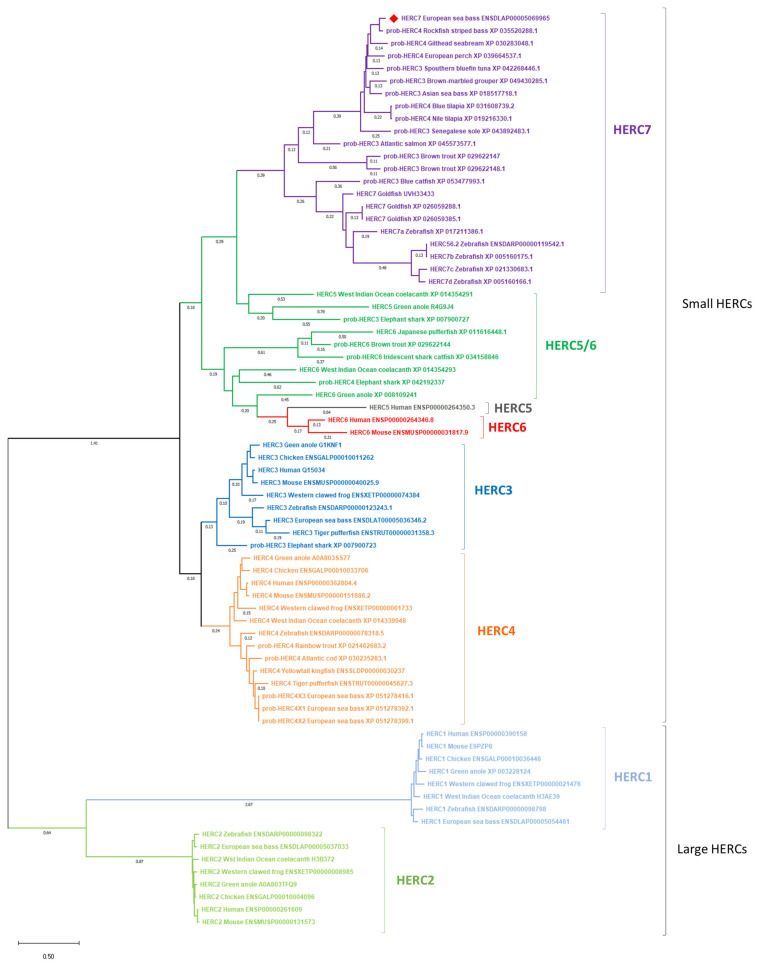
Phylogenetic analysis of European sea bass HERC7. Phylogenetic tree including the European sea bass HERC7 protein was constructed using the Maximum Likelihood method and JTT matrix-based mode, where genetic distances were calculated based on protein differences (p-distance) with pairwise deletion. The tree is drawn to scale, with branch lengths measured in the number of substitutions per site (representing only values over 0.1). Large (HERC1 and HERC2) and small (HERC3, HERC4, HERC5, HERC6, evolutionary intermediate HERC5/6, and fish-exclusive HERC7) HERC proteins are included. Accession numbers are shown. European sea bass HERC7 protein is marked with a red square. Clades corresponding to different HERC proteins are indicated in different colors.

**Figure 2:**
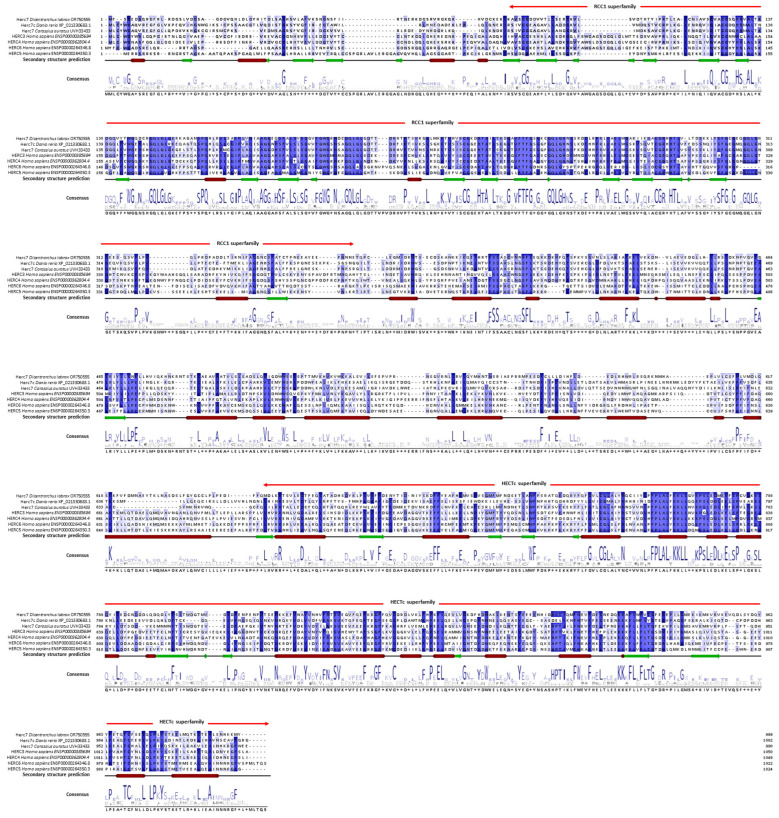
Alignment of the European sea bass HERC7 with its orthologues shows domain and secondary structure conservation. European sea bass HERC7 putative protein sequence was aligned with zebrafish, crucian carp, and human orthologues using Jalview. Numbering was performed according to the European sea bass HERC7. Gaps were introduced to strength the alignment. Residues with consensus higher than 70% are shown in blue gradient and the prediction of secondary structure is detailed, with α-helix as red squares and β-sheet as green arrows. Different regions and superfamilies in the protein are also represented.

We first performed an in silico analysis of the European sea bass HERC7. Thus, from an RNA-seq study we identified a probable *herc4* transcript (DLAgn_00173080) that was up-regulated during the antiviral immune response of European sea bass leucocytes [25], and further confirmed in the genome database (ENSDLAG00005033031). However, thanks to our in silico analysis we propose to rename it as *herc7* (OR750555) accordingly. Thus, European sea bass *herc7* mRNA has 5921 bp encoding for a putative HERC7 protein with 999 amino acids (113 kDa, pI = 6.4). Phylogenetic analysis (Figure 1) revealed that the putative European sea bass HERC7 protein clusters within the small HERC proteins together with the fish-specific HERC7, but in a separated clade from vertebrate HERC3 and HERC4 and mammalian HERC5 and HERC6. Thus, European sea bass HERC1, HERC2, HERC3, and HERC4 are orthologous to those in fish and mammals, forming distinct and independent clades for each of these proteins. It is noteworthy to see that many fish probable HERC proteins grouped in the fish-specific HERC7 clade, probably due to misannotation. Also, evolutionary intermediate independent clades named as HERC5/6 can be observed between fish HERC7 and mammalian HERC5 and 6 (Figure 1). We also aligned the putative European sea bass HERC7 with zebrafish HERC7c, crucian carp HERC7, and hHERC3 to hHERC6 (Figure 2). This alignment, combined with the prediction of the protein’s architecture, revealed consistent conservation within the RCC1 and HECTc domains (Table 1; Figure 2; Supplementary Data S1).

**Figure 3:**
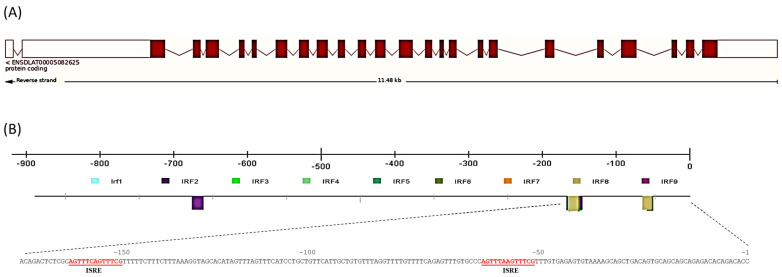
Interferon promoter binding sites are found in the European sea bass *herc7* gene promoter. (**A**) Exon–intron organization of *herc7* gene; (**B**) *herc7* promoter transcription binding factors. IRF, interferon regulatory factors; ISRE, interferon-sensitive regulatory elements.

The highest percentage of identity was obtained against crucian carp (42.73%), whilst hHERC6 showed the lowest percentage (32.66%). In addition, the percent identity of the RCC1 and HECTc superfamily regions were also highest with crucian carp HERC7 (55.48% and 50.00%, respectively) and lowest with hHERC3 (36.83%). These percentages of identity were high even when compared with hHERC4 (31.22% for the full sequence and 42.49% and 38.18% for the RCC1 and HECTc domains, respectively). Secondary and tertiary structure prediction showed several conserved β-sheets in the RCC1 domain and at least one well-conserved one in the HECTc domain (Figure 2, Supplementary Data S1). In addition, the identification of multiple α-helixes between N- and C-terminal sites is highly conserved. At the genomic level, the gene contained 24 exons and 23 introns comprising 11,481 bp (Figure 3A). The promoter analysis revealed the presence of IRF binding sites in the European sea bass *herc7* promoter (Figure 3B). The JASPAR database identified that all the nine IRFs might bind at position −52 and IRF5 to IRF8 might bind at position −152 bp, while only IRF2 is predicted to bind at −670 bp of the European sea bass *herc7* promoter (Figure 3B). In addition, two IFN-sensitive regulatory elements (ISRE) consensus sequence (AGTTTCN2TTTCN) were predicted, at positions −152 (AGTTTCAGTTTCG) and −52 (AGTTTAAGTTTCG) bp, coinciding with the IRF binding sites (Figure 3B). Therefore, it is reasonable to speculate that European sea bass HERC7 could be involved in the IFN response.

### 2.2. herc7 Transcription in Naïve Conditions

**Figure 4:**
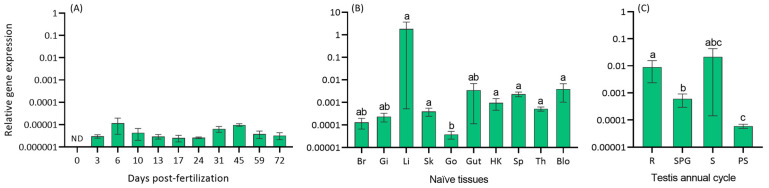
European sea bass *herc7* gene is widely and constitutively expressed in naïve conditions. Transcription of *herc7* in European sea bass (**A**) eggs (0-days post-fertilization (dpf; *n* = 3) and larvae (from 3 to 72 dpf; *n* = 3), (**B**) naïve tissues (*n* = 3), and (**C**) testis of one-year-old specimens within one complete reproductive cycle (*n* = 6). Data are expressed as the mean ± SEM of mRNA transcripts relative to *ef1a* gene expression. Letters denote statistical differences among groups according to one-way ANOVA test (*p* < 0.05). ND, non-detected; Br, brain; Gi, gill; Li, liver; Sk, skin; Go, gonad; HK, head-kidney; Sp, spleen; Th, thymus; Blo, blood; R, resting; SPG, spermatogenesis; S, spawning; PS, post-spawning.

We evaluated the gene expression under naïve conditions by real-time PCR. Regarding ontogeny, our results demonstrated the absence of *herc7* gene expression in newly fertilized eggs (0 days post-fertilization, dpf), with consistent transcriptional levels in larvae from 3 dpf onwards until the end of their expected immunocompetence acquisition, 72 dpf (Figure 4A). Moreover, the *herc7* gene exhibited constitutive and homogeneous expression across most analyzed tissues under naïve conditions (Figure 4B), with the highest and lowest transcription in the liver and gonad, respectively. Transcription of *herc7* was detected during the entire testicular annual cycle with the lowest expression during post-spawning (Figure 4C).

### 2.3. Transcriptional Levels of herc7 Are Modulated by Viruses and Mitogens In Vitro

**Figure 5:**
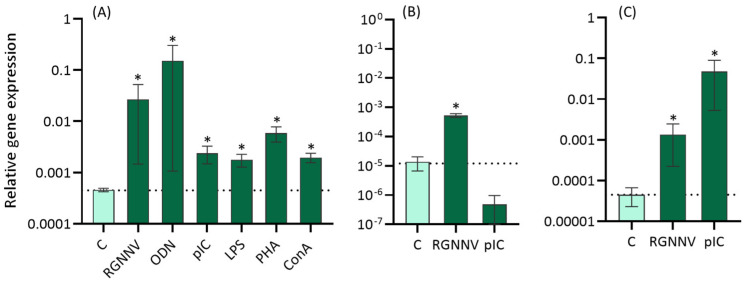
European sea bass *herc7* gene is up-regulated upon different stimuli in vitro. Expression levels of *herc7* gene in European sea bass (**A**) head-kidney leucocytes, (**B**) DLB-1 cell line, and (**C**) testis fragments after 24 h of in vitro treatments studied by real-time PCR. For head-kidney leucocytes: RGNNV (10^7^ TCID_50_ mL^−1^); 50 mg mL^−1^ CpG ODN 1668; 25 mg mL^−1^ pIC; 5 mg mL^−1^ LPS; 10 mg mL^−1^ PHA; or 5 mg mL^−1^ ConA. For DLB-1 and testis fragments: RGNNV (10^7^ TCID_50_ mL^−1^) or 25 mg mL^−1^ pIC. Data are expressed as the mean ± SEM (*n* = 6) of mRNA transcripts relative to *ef1a* gene expression. Asterisks denote statistical differences between treated and control group according to Student’s *t* test (*p* < 0.05). Dotted line: control value.

After the in vitro treatment of head-kidney leucocytes (HKLs) with different immune stimuli (Figure 5), European sea bass *herc7* gene expression was up-regulated by RGNNV, ODN (oligo deoxynucleotide 1668), pIC (polyinosinic:polycytidylic acid), LPS (lipopolysaccharide), PHA (phytohemagglutinin), and ConA (concanavalin A) when compared with control leucocytes (Figure 5A). In contrast, in the DLB-1 cell line, *herc7* gene expression was increased after RGNNV but not upon pIC treatment (Figure 5B). In testis fragments treated in vitro with RGNNV or pIC, the transcriptional levels of *herc7* were increased in both cases (Figure 5C).

### 2.4. RGNNV Blocks the Expression of herc7 Gene in Different Tissues In Vivo

**Figure 6:**
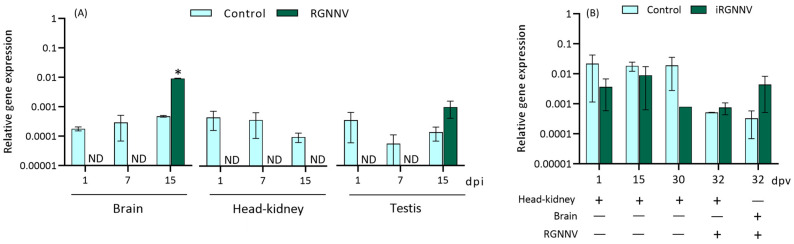
European sea bass *herc7* gene expression is regulated by RGNNV infection in vivo but not by vaccination. Expression levels of *herc7* gene in European sea bass (**A**) brain, head-kidney, and testis after 1, 7 and 15 days of in vivo RGNNV infection (10^6^ TCID_50_ per fish) and (**B**) head-kidney or brain of specimens 1, 15, and 30 days after intraperitoneal injection with PBS (control) or UV-inactivated vaccine (iRGNNV; 10^6^ TCID_50_ per fish) and after 2 days of RGNNV infection (10^6^ TCID_50_ per fish) studied by real-time PCR. Data are expressed as the mean ± SEM (*n* = 5) of mRNA fold increase respect to control samples. Asterisks denote significant differences with controls at each sampling time according to Student’s *t* test (*p* < 0.05). ND: non-detected; dpi: days post-infection; dpv: days post-vaccination; −: absence; +: presence.

After an in vivo challenge with RGNNV (Figure 6A), the results revealed the complete blockage of *herc7* gene expression in the brain and the testis at 1 and 7 dpi, while after 15 dpi it was expressed in both tissues but up-regulated up to control levels in the brain. On the contrary, in the head-kidney, *herc7* expression was blocked at all time-points assayed.

Finally, we analyzed the pattern of expression of the *herc7* coding gene during the vaccination period with an RGNNV inactivated vaccine (iRGNNV) and after 2 days of being challenged with RGNNV (Figure 6B). Our data revealed that neither vaccination nor a subsequent infection altered the transcriptional levels of *herc7* (Figure 6B).

## 3. Discussion

The emergence of -omics techniques has brought to light the significance of HERC proteins in cellular processes, and particularly in the immune response. Consequently, new unknown functions for E3 ubiquitin ligases have come to the frontline. Through the examination of multiple RNA-seq datasets from European sea bass and other fish species, we consistently observed the up-regulation of a probable E3 ubiquitin ligase *herc4* (as annotated in the databases) in the context of RGNNV infections [19,25,26]. This gene was subjected to further and deeper characterization in our study. Whereas full-length reviewed proteins are annotated in public databases for humans, only unreviewed or probable proteins were available for most fish HERC orthologs and paralogs. Although the presence of HERC proteins in teleost fish had previously been suggested to be limited to HERC1 and HERC3 [27], the extensive analysis of fish transcriptomes and genomes has expanded this notion for small HERCs. Thus, the previously identified European sea bass sequence was found to be consistently clustered, forming a clade with the zebrafish and crucian carp HERC7 and with others identified in fish as probable HERC3 or HERC4 and separated from the rest of the small HERC proteins from other vertebrates. Therefore, this European sea bass sequence was renamed as HERC7, as previously suggested [21]. In fact, our phylogenetical study confirms the previous classification into HERC3 (HERC3 and HERC4), HERC5 (mammalian HERC5 and HERC6, and the nonmammalian homologs renamed as HERC5/6s), and fish-exclusive HERC7 subfamilies [21]. These data indicate that HERC7 appeared in cartilaginous fish and was later lost, while the fish HERC5/6 appeared in cartilaginous fish and was also lost in some vertebrate groups but suffered duplication and diversification in others, such as mammals, which was proposed earlier [12]. This divergence could be attributed to adaptive responses to the distinct environmental pressures or ecological niches that fish encountered, leading to the loss of certain HERC proteins while retaining or evolving others. As a matter of fact, genome-scale divergences and species-specific genetic adaptations in various fish lineages have been described [28,29]. It is worthy to note the discrepancies in the nomenclature and annotation of small HERC proteins in fish. RNA-seq data analysis directly annotated HERC1 to HERC4 in fish, unveiling inconsistencies due to incomplete sequences and sometimes non-validated annotations. Thereby, it would be desirable to improve the automatic annotations during -omics studies to avoid misleading assumptions about protein functions, including HERCs in this case.

As a first functional approach in the European sea bass *herc7* gene, we studied the transcriptional levels of this gene in different physiological scenarios. Interestingly, *herc7* gene expression was not detected in 0 dpf eggs, but was later expressed from 3 dpf onwards. These findings indicate that the mRNA of this molecule is not vertically transmitted from the broodstocks to the offspring, as happens with other molecules related to immunity [30]. However, it is expressed very early in the development as also described in European sea bass for several innate immune genes [31]. Once juveniles, the *herc7* gene is expressed in all tissues analyzed, including those related with immunity, metabolism, the nervous system, or reproduction, pointing to its involvement in a wide range of biological functions as addressed for mammalian HERC proteins (for a review, see [6]). Regarding reproduction, our data showed that the highest levels reached in the testis corresponded with those of the spawning stage as also described for other immune molecules [32]. It is explained by the need to protect the sperm storage in the testicular lumen from opportunistic pathogens or due to the cellular reorganization and differentiation that occurs during spermatozoa production and maturation [32]. In fact, and in contrast to what happens with other immune molecules previously analyzed [32], the levels of transcription of *herc7* in the testis at the resting stage are also very high, coinciding with a very intensive cell proliferation and apoptosis that lead to tissue renewal as described in several fish species [33,34]. Thus, our data point to a role for *herc7* in testis related to tissue renewal and cell differentiation, which deserves further investigations. Supporting this idea, hHERC5 is highly expressed in the testis, and a clear role in spermatogenesis and male infertility has been linked to hHERC4 and mHERC6 [27,35,36]. Considering that HERC7 is a unique protein in fish, the analysis of the expression pattern of the *herc7* gene may elucidate differences in reproductive strategies compared to other vertebrates from an evolutionary perspective, which deserves further investigation.

hHERC5’s and fish HERC7’s functions have been clearly linked to the immune response, and particularly to antiviral immunity since viral infection resulted in the up-regulation of both hHERC5 and fish HERC7 mRNA [13,21]. However, their biological functions seem to be contradictory. hHERC5 increases ended in improved IFN response and viral clearance [9,13,15,16,17], while fish HERC7 overproduction led to the elimination of some antiviral mediators and higher viral replication [21,22]. To test HERC7’s potential role in sea bass immunity, we in vitro-stimulated sea bass HKLs with RGNNV and different immune stimuli and mitogens. The expression of the *herc7* gene was significantly up-regulated in sea bass HKLs following stimulation with B and T cell mitogens, RGNNV, and pIC, suggesting a role in the inflammatory and antiviral responses. In fact, hHERC5 was considered a late inflammatory protein in humans that is increased by pro-inflammatory cytokines (IL-1β and TNFα) and LPS through the NF-κB pathway [37]. This could be occurring in the European sea bass HKLs stimulated by mitogens. In addition, hHERC5 is also increased by the IFN stimulation of human cells [10], as in our HKLs stimulated with RGNNV or pIC, which induces the IFN pathway and links fish *herc7* with the antiviral response. Unfortunately, no studies in fish have evaluated the regulation of *herc* genes in fish leucocytes upon stimulation.

Based on previous studies and our data in HKLs, we tried to study the correlation between the European sea bass *herc7* gene and the antiviral response. In a first attempt, we evaluated it in the sea bass brain DLB-1 cell line and primary cultures of testis fragments as RGNNV has been described to colonize this tissue and elicit a clearly different immune response than in other tissues [32]. In both cases, RGNNV infection up-regulated the *herc7* mRNA in line with prior studies in virus-infected fish cell lines [21,22]. Afterwards, we evaluated the impact of the viral infection in vivo. Strikingly, RGNNV infection completely blocked the expression of the *herc7* coding gene in the head-kidney, brain, and testis of European sea bass up to 7 dpi, to be later up-regulated or basally expressed in the brain and testis, respectively. However, our data also showed that in some cases, the blockage observed in the NNV-infected brain does not occur at early times of infection, as revealed by the unvaccinated and infected fish from our vaccination study [38]. This data suggests that *herc7* gene regulation might be related to the virulence of the infection as in the in vivo infection the mortalities appeared 2 days earlier than in the vaccination study [32,38]. In this regard, RGNNV infection in the brain resulted in a very high production of viral particles and the inhibition of most molecules involved in the type-I IFN pathway during the early stages of infection [32]. Thus, the regulation of sea bass *herc7*- and IFN-related genes is somehow parallel during RGNNV infection. In addition, the low RGNNV replication occurring in the DLB-1 cell line and testis in vivo could be due to the high type-I IFN response elicited from the first day onwards in those tissues [26,32,39], inducing *herc7* transcription at different time points as our data revealed. Taking all this into account, our data might indicate that depending on the virulence of the infection and the IFN response elicited [32], *herc7* transcription is altered. Additionally, *herc7* transcription in vaccinated sea bass specimens was unaltered like that of the IFN-related genes [38]. In fact, the vaccine blockage of RGNNV induced down-regulation of the IFN-related gene expression, resulting in a decrease in viral replication (more than 30-fold) and an increase in the protection rate (59.7% survival) in vaccinated fish. As our data indicate that *herc7* expression is higher in those tissues where RGNNV replication is low and the IFN pathway is expressed, it seems reasonable to hypothesize that IFN mediators, and not viral genome or proteins, activate the transcription of *herc7*, as suggested by the presence of IRF and ISRE binding sites in the *herc7* gene promoters of sea bass, crucian carp, and zebrafish ([21,22] and this study), and in turn HERC7 might be involved in viral protein degradation. However, the overexpression of zebrafish HERC7 led to the suppression of the type-I IFN pathway response through targeting STING, MAVS, and IRF7 [22], which in turn increased viral replication. Thus, we could also assume that this HERC7 protein would mediate the elimination of IFN-related proteins through ISGylation to control the exacerbation of the antiviral response. Regarding the interaction between fish HERC7 and viral proteins, unfortunately, the studies performed in fish only focused on HERC7’s interaction with some IFN mediators [21,22] but not with the viral proteins and our study cannot elucidate this issue. However, the coelacanth HERC5, which is clearly clustered within the fish HERC7 clade as seen here and in previous studies [22], was able to reduce the amounts of simian virus immunodeficiency virus proteins but not those of human retrovirus. Therefore, further studies under natural or more reliable infection models would shed some light on the precise tuning between IFN, HERC7, and viral proteins in fish, which seems to be crucial for host immunity and resistance.

To sum up, our in silico study greatly supports that the identified gene would encode the putative HERC7 protein of European sea bass, with conserved protein domains and also probably conserved functions. The up-regulation of *herc7* upon in vitro stimulation with pathogens, immune *stimuli*, and mitogens suggests its involvement in the immune response against viruses. The role in the antiviral response is also predicted by the conserved presence of IRF and ISRE binding sites in the promoter. In fact, our findings revealed that the viral replication levels, the transcription of IFN-pathway molecules, and the *herc7* gene expression could be finely tuned up depending on the tissue infected. However, HERC7’s interaction with viral proteins is not clear in fish, raising doubts about the direct antiviral role of fish HERC7 and underscoring the need for extended protein and functional research to fully elucidate its roles in the context of viral infections in fish.

## 4. Materials and Methods

### 4.1. Bioinformatics and Genetic Analysis

A probable herc4 transcript was identified in an RNA-seq study carried out in our laboratory with European sea bass (*Dicentrarchus labrax*) leucocytes [25], whose sequence was further confirmed within the European sea bass genome project “http://seabass.mpipz.mpg.de/” (accessed on 27 March 2024). Protein sequences were used for multiple sequence alignments using BLAST/BLAT. Calculations of percent identity and secondary structure prediction were performed with Jalview [40]. Protein domain architecture was searched in NCBI “https://www.ncbi.nlm.nih.gov/” (accessed on 2 April 2024) and InterPro “https://www.ebi.ac.uk/interpro/” (accessed on 2 April 2024) databases. Three-dimensional modelling of protein structure was conducted using the SwissModel tool from Expasy ”https://swissmodel.expasy.or/” (accessed on 2 April 2024). Analysis of the transcription factor binding sites in the promoter sequence was performed using CiiiDer software “http://ciiider.com/” (accessed on 15 April 2024). Evolutionary analyses were conducted in MEGA11 (MEGA, Philadelphia, PA, USA) [41] using the Maximum Likelihood method and JTT matrix-based model [42]. Based on the molecular analysis results, the gene was renamed to herc7.

### 4.2. Experimental and Sampling Procedures

Considering the 3Rs principle we evaluated the herc7 gene’s expression levels in European sea bass cDNA samples previously obtained and tested [32,43,44], and in DLB-1 cell line as summarized below.

For ontogenetic study purposes, 3 pools of eggs (500 mg each) at 0 days post-fertilization (dpf) and 3 pools of whole larvae (300 mg each) at 3, 6, 10, 13, 17, 24, 31, 45, 59, and 72 dpf were used [43]. For the tissue expression study, tissues (brain, gill, liver, skin, gonad, gut, head-kidney, spleen, thymus, and blood) from 3 naïve fish specimens were sampled independently [32]. Testis of 1-year-old sea bass males were used for the analysis of the expression throughout the reproductive cycle [32].

For the in vitro stimulation of HKLs (*n* = 5), 10^7^ HKLs mL^−1^ were incubated in flat-bottomed 48-well microtiter plates (ThermoFisher Scientific, Waltham, MA, USA) at 22 °C during 24 h with culture medium alone (control), 5 μg mL^−1^ lipopolysaccharide (LPS; MilliporeSigma, St. Louis, MO, USA), 10 μg mL^−1^ phytohemagglutinin (PHA; MilliporeSigma), 5 μg mL^−1^ concanavalin A (ConA; MilliporeSigma), 50 μg mL^−1^ synthetic unmethylated cytosine-phosphodiester guanosine oligo deoxynucleotide 1668 (CpG ODN 1668; sequence 5′-TCCATGACGTTCCTGATGCT-3′; Eurogentec, Seraing, Belgium), 25 μg mL^−1^ polyinosinic:polycytidylic acid (pIC; MilliporeSigma), or 10^6^ TCID_50_ mL^−1^ of RGNNV (strain 411/96) [44]. For the in vitro stimulation of the testis, we used 1 mm^3^ fragments of testis (*n* = 3) to culture them with culture medium alone (control), 10^7^ TCID_50_ mL^−1^ of RGNNV, or 25 μg mL^−1^ of pIC at 25 °C during 24 h [32]. Finally, DLB-1 cell line was treated with 10^6^ TCID_50_ mL^−1^ of RGNNV or 25 μg mL^−1^ of pIC in triplicate at 25 °C during 24 h. Before processing for gene expression analysis, HKLs, DLB-1 cells, and testis fragments were washed with 0.01 M phosphate-buffered saline (PBS).

For the in vivo RGNNV infection, fish at resting reproductive stage were mock- (control) or RGNNV-infected (10^6^ TCID_50_ RGNNV per fish) in a total volume of 100 μL per fish. Fish (*n* = 5 fish group-1 time-1) were sampled 1, 7, or 15 days post-infection (dpi) and brain (target tissue of RGNNV), head-kidney, and testis tissues were sampled [32].

Finally, the vaccination of European sea bass juveniles was carried out intraperitoneally with culture medium alone (control) or an UV-inactivated RGNNV (iRGNNV; 10^7^ TCID_50_ per fish) [38]. After 30 days post-vaccination (dpv), fish were challenged intramuscularly with 10^6^ TCID_50_ RGNNV per fish. Samples of head-kidney were obtained at different time-points during the vaccination period (*n* = 6 fish group^−1^ time^−1^; 1, 15, and 30 dpv) and head-kidney and brain samples were collected at 2 dpi (*n* = 6 fish; corresponding to 32 dpv) [38].

Samples from all experimental procedures were immediately immersed in Trizol^®^ Reagent (Thermo Fisher Scientific, Carlsbad, CA, USA) after sampling and stored at −80 °C.

### 4.3. Real-Time PCR for Gene Expression Analysis

Total RNA was isolated from TRIzol^®^ Reagent-frozen samples following the manufacturer’s instructions. Then, 1 μg of total RNA was treated with DNAse I (Promega, Madison, WI, USA) to remove genomic DNA and the first strand of cDNA synthesized by reverse transcription using the Superscript IV (Thermo Fisher Scientific).

To analyze the expression levels of European sea bass *herc7*, qPCR reactions were performed with an ABI PRISM 7500 instrument (Thermo Fisher Scientific) using SYBR Green PCR Core Reagents (Thermo Fisher Scientific). Reaction mixtures were incubated at 95 °C for 10 min; followed by 40 cycles of 15 s at 95 °C, 1 min at 60 °C; and finally 15 s at 95 °C, 1 min at 60 °C, and 15 s at 95 °C. For each RNA, the relative gene expression, expressed as 2^−ΔCt^ [45], was calculated by subtracting the elongation factor 1 alpha (*ef1a*) gene expression, as endogenous control, Ct value from the target gene Ct value. This relative gene expression was plotted in different graphs. The primers used were designed specifically using the Oligo Perfect software tool “https://apps.thermofisher.com/apps/olilgoperfect” (accessed on 5 December 2022) and are shown in Supplementary Data S2. Before the experiment, the specificity of each primer pair was studied using positive and negative samples. A melting curve analysis of the amplified products validated the primer for specificity at each use. Negative controls with no template were always included in the reactions.

### 4.4. Statistical Analysis

All data are presented as mean ± standard error of the mean (SEM). Results were analyzed by one-way ANOVA test followed by Tukey’s post hoc analysis to examine the differences between naïve tissues, eggs, and larvae at different time-points of their development or in testis between different reproductive stages (*p* < 0.05). The Student *t* test was used to denote statistical differences between infected/vaccinated and control groups (*p* < 0.05). A non-parametric Kruskal–Wallis test, followed by a multiple comparison test, was used when data did not meet parametric assumptions. All statistical analyses were conducted using IBM SPSS20 software.

## 5. Conclusions

Our in silico study greatly supports that the identified gene would encode the putative HERC7 protein of European sea bass, with conserved protein domains and probably conserved functions. The up-regulation of *herc7* upon in vitro stimulation with pathogens, immune stimuli, and mitogens suggests its involvement in the immune response against viruses. The role in the antiviral response is also predicted by the conserved presence of IRF and ISRE binding sites in the promoter. In fact, our findings revealed that the viral replication levels, the transcription of IFN-pathway molecules, and the *herc7* gene expression are finely tuned up depending on the tissue infected. However, HERC7’s interaction with viral proteins is not clear in fish, raising doubts about the direct antiviral role of fish HERC7 and underscoring the need for extended research to fully elucidate its functions in the context of viral infections in fish.

## Figures and Tables

**Table 1:** Identity percentage of European sea bass HERC7 with zebrafish HERC7c, crucian carp HERC7, and human hHERC3 to 6.

Sequence	Percent Identity (%)			Reference
	Full Sequence	RCC1 Superfamily	HECTc Superfamily	
European sea bass HERC7	100.00	100.00	100.00	This work; OR750555
Zebrafish HERC7c	38.35	51.75	36.02	[22]
Crucian carp HERC7	42.73	55.48	50.00	[21]
Human HERC3	33.68	42.12	36.83	ENSP00000385684
Human HERC4	31.22	42.49	38.18	ENSP00000362804.4
Human HERC5	34.50	39.24	41.14	ENSP00000264346.8
Human HERC6	32.66	40.70	40.80	ENSP00000264350.3

## Data Availability

Data are contained in this manuscript.

## Supplementary Materials

The following supporting information can be downloaded at https://www.mdpi.com/article/10.3390/ijms25147751/s1.
