# Supplementary material for: Fish HERC7: Phylogeny, Characterization, and Potential Implications for Antiviral Immunity in European Sea Bass
Source: Int J Mol Sci. 2024 Jul 15;25(14):7751. doi: 10.3390/ijms25147751 (PMC11277259; doi:10.3390/ijms25147751)
Supplement: Supplementary file 1 [file ijms-25-07751-s001.zip › Supplementary Data S1.pptx]

## Slide 1
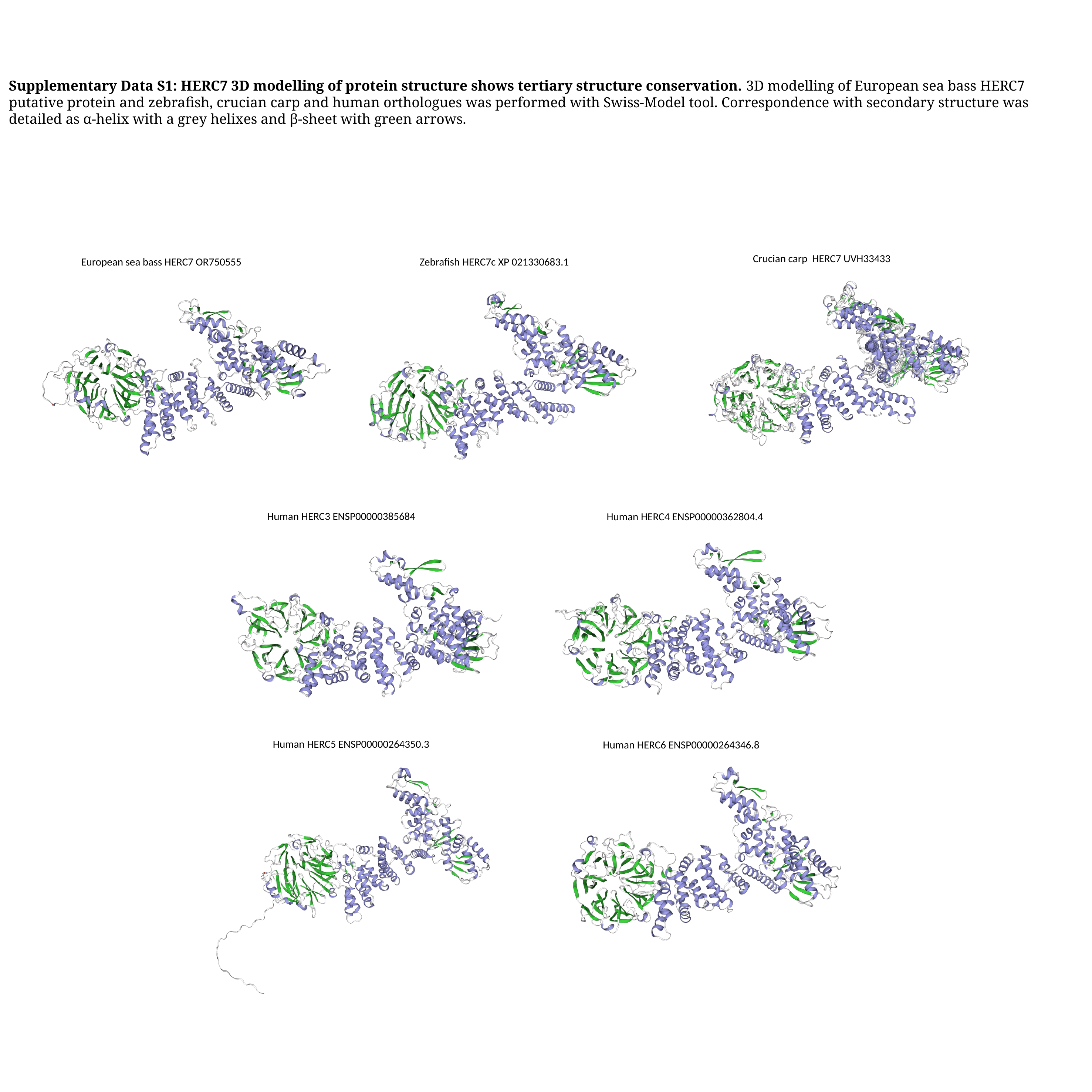

Supplementary Data S1: HERC7 3D modelling of protein structure shows tertiary structure conservation. 3D modelling of European sea bass HERC7 putative protein and zebrafish, crucian carp and human orthologues was performed with Swiss-Model tool. Correspondence with secondary structure was detailed as α-helix with a grey helixes and β-sheet with green arrows.
Crucian carp HERC7 UVH33433
European sea bass HERC7 OR750555
Zebrafish HERC7c XP 021330683.1
Human HERC3 ENSP00000385684
Human HERC4 ENSP00000362804.4
Human HERC6 ENSP00000264346.8
Human HERC5 ENSP00000264350.3
