# Supplementary material for: Fish HERC7: Phylogeny, Characterization, and Potential Implications for Antiviral Immunity in European Sea Bass
Source: Int J Mol Sci. 2024 Jul 15;25(14):7751. doi: 10.3390/ijms25147751 (PMC11277259; doi:10.3390/ijms25147751)
Supplement: Supplementary file 1 [file ijms-25-07751-s001.zip › Supplementary Data S2.pdf]

Supplementary Data S2: Primers used for real-time PCR analysis.

**Protein name**	**Gene name**	**Accession number**	**Sequence (5' – 3')**	
E3 ubiquitin protein ligase 7	*herc7*	OR750555	F<br>R	AAGCAGAGCACAATGGAGCT<br>GATCGCTGACAGAGATCGCA
Elongation factor 1 alpha	*ef1a*	AJ866727	F<br>R	CGTTGGCTTCAACATCAAGA<br>GAAGTTGTCTGCTCCCTTGG
